# Supplementary material for: Effectiveness of transcranial direct current stimulation on hand dexterity in stroke patients: a protocol for a systematic review and meta-analysis
Source: BMJ Open. 2022 Feb 15;12(2):e056064. doi: 10.1136/bmjopen-2021-056064 (PMC8852708; doi:10.1136/bmjopen-2021-056064)
Supplement: Supplementary data [file bmjopen-2021-056064supp001.pdf]

SUPPLEMENTARY FILE 1:
Database search strategy for MEDLINE

1. Cerebrovascular accident
2. Cerebrovascular disorder
3. Cerebr*
4. Stroke
5. Poststroke
6. Post-stroke
7. CVA
8. Hemiplegi*
9. Hemipare*
10. Or/1,2,3,4,5,6,7,8,9
11. Transcranial direct current stimulation
12. TDCS
13. Brain stimulation
14. Or/11,12,13
15. Physiotherapy
16. Physical therapy
17. Occupational therapy
18. Rehabilitation
19. Motor training
20. Motor*
21. Exercise therapy
22. Constraint induced movement therapy
23. Modified constraint induced movement therapy
24. Or/15,16,17,18,19,20,21,22,23
25. Upper extremit*
26. Upper limb*
27. Arm
28. Hand
29. Or/25,26,27,28
30. Motor function
31. Function*
32. Functional outcome measure*
33. Outcome measure*
34. Dexterity
35. Dexter*
36. Manual dexterity
37. Fine
38. Gross
39. Or/30,31,32,33,34,35,36,37,38
40. Randomized controlled trial
41. RCT
42. Random*
43. Controlled clinical trial
44. Control*
45. Trial

46. Double-blind*
47. Double*
48. Sham-controlled
49. Sham*
50. Placebo-controlled
51. Placebo*
52. Groups
53. Allocate*
54. Assign*
55. Or/40,41,42,43,44,45,46,47,48,49,50,51,52,53,54
56. Child*
57. Cerebral palsy
58. Lower limb*
59. Lower extremit*
60. Leg
61. TMS
62. rTMS
63. Transcranial magnetic stimulation
64. Or/56,57,58,59,60,61,62,63
65. And/10,14,24,29,39,55
66. 65 NOT 64
67. Limit to human
