# Supplementary material for: Effectiveness of transcranial direct current stimulation on hand dexterity in stroke patients: a protocol for a systematic review and meta-analysis
Source: BMJ Open. 2022 Feb 15;12(2):e056064. doi: 10.1136/bmjopen-2021-056064 (PMC8852708; doi:10.1136/bmjopen-2021-056064)
Supplement: Author's manuscript [file bmjopen-2021-056064.draft_revisions.pdf]

BMJ Open

BMJ Open is committed to open peer review. As part of this commitment we make the peer review history of every article we publish publicly available.

When an article is published we post the peer reviewers' comments and the authors' responses online. We also post the versions of the paper that were used during peer review. These are the versions that the peer review comments apply to.

The versions of the paper that follow are the versions that were submitted during the peer review process. They are not the versions of record or the final published versions. They should not be cited or distributed as the published version of this manuscript.

BMJ Open is an open access journal and the full, final, typeset and author-corrected version of record of the manuscript is available on our site with no access controls, subscription charges or pay-per-view fees (http://bmjopen.bmj.com).

If you have any questions on BMJ Open's open peer review process please email info.bmjopen@bmj.com

The effectiveness of transcranial direct current stimulation on hand dexterity in stroke patients: a protocol for a systematic review and meta-analysis.

Journal:	*BMJ Open*
Manuscript ID	bmjopen-2021-056064
Article Type:	Protocol
Date Submitted by the Author:	03-Aug-2021

Complete List of Authors:	Alabdulaali, Lulwa; University of Birmingham; Imam Abdulrahman Bin Faisal University,<br>Hickman, Lydia; University of Birmingham, School of Psychology<br>Jenkinson, Ned; University of Birmingham, School of Sport, Exercise and Rehabilitation Sciences; University of Birmingham, Centre for Human Brain Health

Keywords:	REHABILITATION MEDICINE, Stroke < NEUROLOGY, Neurology < INTERNAL MEDICINE

TITLE: The effectiveness of transcranial direct current stimulation on hand dexterity in stroke patients: a protocol for a systematic review and meta-analysis.

AUTHORS

First author (corresponding author): Lulwa Alabdulaali

Institutional affiliation:

1- School of Sport, Exercise and Rehabilitation Sciences, University of Birmingham, UK
2- Department of Physical Therapy, College of Applied Medical Sciences, Imam Abdulrahman Bin Faisal University, Dammam, Saudi Arabia

e-mail address: Laa886@student.bham.ac.uk

Mailing address: University of Birmingham, Edgbaston, Birmingham, B15 2TT, UK

Second author: Lydia Hickman

Institutional affiliation:

1. School of Psychology, University of Birmingham, UK

e-mail address: Lxh856@student.bham.ac.uk

Third author: Ned Jenkinson

Institutional affiliation:

1. School of Sport, Exercise and Rehabilitation Sciences, University of Birmingham, UK
2. MRC-Versus Arthritis Centre for Musculoskeletal Ageing Research, University of Birmingham, UK
3. Centre for Human Brain Health, University of Birmingham, UK

e-mail address: n.jenkinson@bham.ac.uk

WORD COUNT: 3160

For peer review only

ABSTRACT

Introduction

Hand dexterity is the ability to execute the skilful movements using the hand and fingers. It is commonly impaired post-stroke resulting in a profound deterioration in the quality of life for stroke patients. Transcranial direct current stimulation (tDCS) is a form of non-invasive brain stimulation, which has gained a popularity as an adjunct therapy in recovering motor dysfunction post-stroke. Promising results have been gained from applying tDCS in combination with motor rehabilitation, however, the outcome of tDCS on the upper limb motor function post-stroke has been varied. Different results are potentially related to the discrepancy of the area of brain stimulation. Also, the dissociated recovery pattern of the proximal and distal segments of the upper limb could contribute to the varied results. Therefore, we aim to enhance the application of tDCS to improve its effectiveness in recovering hand dexterity through testing our hypothesis that stimulating the primary motor cortex could improve fine dexterity more than gross dexterity.

Methods and analysis

This protocol has been reported according to Preferred Reporting Items for Systematic Review and Meta-Analyses Protocols guidelines. CENTRAL, MEDLINE, EMBASE, SCOPUS and Web of Science databases will be searched with no restriction in language and publication date. The selected studies will be randomised controlled trial investigating the effect of tDCS alone or in combination with motor rehabilitation in improving hand dexterity of stroke patients with upper limb hemiparesis. The outcomes of interest are fine and gross hand dexterity measures. Two independent reviewers will assess the eligibility of the study, extract data and appraise the methodological quality. The data will be pooled in a meta-analysis if applicable or interpreted narratively. Grading of Recommendations, Assessment, Development and Evaluation (GRADE) approach will be used to assess the overall quality of evidence for the fine and gross dexterity measures.

Ethics and dissemination

Ethical approval is not required for this study. The dissemination plan is to publish the results in a peer-review journal and presenting results in a conference.

Registration details

The Protocol is registered in the International Prospective Register of Systematic Reviews database, under PROSPERO CRD42021262186.

For peer review only

STRENGTHS AND LIMITATIONS OF THIS STUDY

- This study will focus on the difference in the recovery between fine and gross hand dexterity.
- This study potentially enhancing the application of tDCS.
- This study could optimise the therapeutic benefit of tDCS.
- The search strategy is not limited to publication date or language.
- This review will be limited to published studies.

INTRODUCTION

Stroke is a common cause of disability in the United Kingdom.[1] Upper limb hemiparesis is a common impairment post-stroke, with seventy-six percent of stroke patients suffering from upper limb hemiparesis.[2] The clinical presentation of upper limb hemiparesis is muscle weakness and poor dexterity on the side of the body opposite of the side of brain lesion.[3 4] Dexterity refers to 'the fine, voluntary movements used to manipulate small objects during a specific task, as measured by the time required to complete the task'.[5(p209)] There are two types of dexterity: fine and gross. Fine dexterity is the ability to perform a delicate manipulation of very small object by precise movements of the fingertips.[6] Gross dexterity is the ability to make a manipulation of large object by coordinated arm-hand movement.[6] The majority of daily activities, such as self-care, dressing and eating, require a combination of fine and gross dexterity in combination with other upper limb movements.[7] Therefore, maintaining hand dexterity is an important determinant of quality of life for stroke patients.[8]

However, stroke patients with upper limb hemiparesis demonstrate dexterity dysfunction. They perform movements in a slow, uncoordinated manner because their hand movements lack accuracy and proper finger sequencing.[3 9] Likely more than half of the stroke patients have persistent hand dexterity deficiency at 6 months post stroke. Kwakkel and colleagues conducted a prospective cohort study,[2] in which they recruited 102 stroke patients from 7 different hospitals. All the patients were enrolled in an intensive rehabilitation program, with the study procedure starting within two weeks of stroke occurrence. Periodic follow up assessments of the upper and lower limbs function were conducted. For hand dexterity they used the Action Research Arm Test (ARAT), which is a reliable and valid tool for assessing hand

For peer review only

function.[10 11] At first assessment, no dexterous hand movement was reported in the ARAT. At 6 months, 38% showed improvement in functional ability, while 62% persisted with loss of hand dexterity.[2]

Stroke rehabilitation involves assessing and improving hand dexterity in stroke patients in order to improve upper limb function.[12] Many functional assessment tools have been established to measure the level of upper limb motor function. These tools include tests for muscle strength and gross and fine hand dexterity. Examples of such tests include the Fugl Meyer Test,[13] Wolf Motor Function Test[14] and Action Research Arm Test.[10] There has also been considerable interest in examining the efficacy of different techniques to enhance upper limb function post-stroke, with some positive results described with a variety of methods as repetitive task training,[15] constraint induced movement therapy,[16] and non-invasive brain stimulation.[17 18]

A recent development has been the use of transcranial direct current stimulation (tDCS), a form of non-invasive brain stimulation. tDCS device includes a portable electrical current generator and two electrodes: cathode and anode, and works by delivering a low intensity constant electrical current to the brain that alters the cortical excitability.[19] It is a neuromodulatory technique that has gained popularity for use in recovering motor dysfunction and cognitive impairment.[12] It has been used to improve motor function among stroke patients, Parkinson's disease patients and old people.[17 20-22] Increased brain plasticity is thought to be the underlying mechanism of functional improvement.[19] Due to tDCS' promising effectiveness, cost-effectiveness, ease of application, safety, and tolerability, recent years have seen increased investigation of use.[12 19 23 24]

Studies exploring the effect of stroke on the brain using functional magnetic resonance imaging have observed decreased neuronal activity of the ipsilesional side few days post stroke.[25] This reduced activity, in turn decreases interhemispheric inhibition from the ipsilesional to the contralesional side, leading to an increase in contralesional neuronal activity, which increases interhemispheric inhibition from the contralesional to the ipsilesional side. This abnormal increase of interhemispheric inhibition likely restricts voluntary movement and therefore adversely affects motor recovery.[24-26] Given these

For peer review only

abnormal patterns of activity, tDCS has been used in an attempt to produce focal changes in neuronal activity to rebalance brain activity between the two hemispheres. The focal changes are either depolarising (diminishing the contralesional excitability), hyper-polarising (increasing the ipselisional excitability), or both, depending on the tDCS montage.[27 28]

Researchers have investigated factors to enhance the application of tDCS to increase its effectiveness, for example, the area of brain stimulation and the orientation of the electrodes.[19] There are three montages of tDCS application described in the literature in improving upper limb motor recovery post troke. The first montage is anodal tDCS. In this montage, the anodal electrode that produces a polarising effect is placed on the ipsilesional primary motor cortex (PMC), and the cathode electrode is used as a return electrode to close the electric circuit. It is placed on the contralesional supraorbital region.[12 19] In the cathodal tDCS montage, the cathode electrode that produces the depolarising effect is placed on the contralesional PMC. The anode electrode serves as the return electrode and is placed on the ipsilesional supraorbital region.[12 19] In the bilateral tDCS montage, the anodal electrode is placed over the ipsilesional PMC and the cathode is placed over the contralateral PMC.[12] Moreover, in controlled studies, tDCS can be applied as a sham stimulation. The sham tDCS is the method to blind the control group. The participant receives a very weak and short stimulating current, at a level below that which could produce any stimulation.[29]

tDCS has shown potential effectiveness in improving upper limb function post stroke when conducted both alone[22] or in combination with motor rehabilitation.[18 30] Several randomised controlled studies investigated the augmentation of the rehabilitation plasticity effect through conjunct therapy.[18 30 31] Conjunct therapy involves tDCS and motor rehabilitation. These studies found a superior beneficial effect of conjunct therapy in upper limb recovery compared to rehabilitation only. However, the efficacy of tDCS in improving hand function has been showed to be varied.[20] Discrepancies in results could be related to heterogeneity in the application of tDCS in the context of current parameters, electrodes montage and brain stimulating area. Other factors may be related to the characteristics of stroke patients, such as stroke phase and the extent and the area of the lesion.[12 32]

For peer review only

Additionally, the dissociated recovery pattern of the upper limb post-stroke could contribute to the varied results seen using tDCS. It has been observed that the proximal part of the upper limb is recovered better than the distal part in response to either tDCS[17] or comprehensive motor rehabilitation for the whole upper limb.[3 33] The study done by Hummel, Celnik, Giraux et al.,[17] investigated the effect of stimulating the primary motor cortex using tDCS on upper limb function, using the Jebsen–Taylor Hand Function Test as an outcome measure. It includes subtests for both fine and gross hand movements. Hummel and colleagues found that the fine movements, like picking up paper clips, are more improved than gross movement like moving cans.

Although differentiation in upper limb recovery was explained in the literature by gradient in the paresis from proximal to distal segments, other studies explored that the paresis affects the whole upper limb equally in acute, subacute and chronic stroke patients.[3 33] It has been suggested that differentiation in the recovery could be related to the cortical control of the upper limb. In monkeys, there is a gradient in the PMC control of the upper limb. The distal segment receives higher input than the proximal part.[34 35] It has been proposed, in stroke patients, that other neural pathways contribute to controlling the proximal part, therefore, contribute to its recovery.[36]

RATIONALE

Given the essential role of the upper limbs in daily life, the recovery of upper limb motor function post stroke is an important issue for stroke rehabilitation community.[12] Improving upper limb function is a challenge for clinicians and researchers because of varying result. The variation in the findings has led to incomplete understanding of upper limb recovery post stroke. Several systematic reviews were conducted to investigate the effect of tDCS on upper limb motor function.[20 27 37-40] The previous reviews assessed the impact of tDCS on improving upper limb functional outcomes. However, they considered the improvement in the upper limb with no differentiation between fine and gross hand dexterity.

We will conduct this systematic review and meta-analysis to investigate the enhancement of tDCS application therefore optimising its therapeutic benefit in upper limb motor recovery post-stroke. Specifically, we will investigate our hypothesis that stimulating the primary

For peer review only

motor cortex using tDCS might improve the fine hand dexterity more than the gross dexterity. We will look for the difference in the recovery between fine and gross hand tasks.

OBJECTIVES

This research aims to determine whether there is a difference in the tDCS effect between fine and gross hand dexterity of stroke patients with upper limb paresis.

Specific objectives are:

1. To evaluate the effectiveness of tDCS on fine hand dexterity of stroke patients with upper limb hemiparesis.
2. To evaluate the effectiveness of tDCS on gross hand dexterity of stroke patients with upper limb hemiparesis.
3. To compare the effect of tDCS between fine and gross hand dexterity.
4. To investigate the impact of patient characteristics on the effect of tDCS.

METHODS AND ANALYSIS

This protocol has been reported according to Preferred Reporting Items for Systematic Review and Meta-Analyses Protocols (PRISMA-P) guidelines.[41]

Eligibility criteria

Types of studies

Inclusion criteria

- Randomised controlled double or single-blinded trials. Using either parallel or crossover design.
- English and non-English studies.
- Full-text studies.
- Published studies.

Exclusion criteria

- Non-randomised clinical trial.
- Unpublished studies.
- Conference abstract.

For peer review only

Types of participants

The participants needed to be:

- Stroke patients aged 18 and above, both male and female with upper limb hemiparesis.
- Diagnosed with ischemic or haemorrhagic stroke, either cortical or subcortical.
- In the sub-acute (2 to 12 weeks post-stroke) or chronic phase (after 12 weeks post-stroke) when received the intervention.
- Suffering from mild or moderate or severe upper limb impairment.

Types of intervention

Intervention group

The participants have received one or multiple sessions of tDCS that conducted alone or with motor rehabilitation. The tDCS either applied before or simultaneously with motor rehabilitation. The tDCS stimulated the primary motor cortex through one of the following montages: anodal, cathodal and bilateral. The motor rehabilitation includes any technique of motor training for upper limb.

Control group

The participants have received one or multiple sessions of sham tDCS or motor rehabilitation or sham tDCS plus motor rehabilitation.

Types of outcome measures

The outcomes of interest are as follow:

- Validated functional measures for fine hand dexterity including Purdue Pegboard Test (PPT) or any other measure.
- Functional measures for gross hand dexterity including Box and Blocks Test (BBT) or any other measures.

Information sources

We will conduct database and non-database searches with no restriction on publication date.

1. Database search

The following databases will be searched:

For peer review only

CENTRAL (Cochrane Central Register of Controlled Trials).

MEDLINE (Ovid).

EMBASE (Ovid).

SCOPUS.

Web of Science.

2. Non-database search

We will review the reference list of the included studies.

Search strategy

An example of search strategy for searching MEDLINE is available as an online supplementary file 1.

Study records

Data management

The search records from the selected databases including the study title, abstract will be exported to a reference management software (EndNote X9). The EndNote X9 will organise the records and recognise the duplicate studies.

Selection process

After removing the duplicate reports using EndNote X9, two independent reviewers will assess the eligibility of the study and discard the irrelevant ones. Initially, the two reviewers will review the studies title and abstract according to the inclusion criteria and exclude the irrelevant studies. Then the full text will be obtained and screened for those studies that potentially meet the inclusion criteria and if more details are required to confirm study exclusion. The two reviewers will decide about the study inclusion and will make discussion if there is disagreement. The third reviewer will make the final decision if the two reviewers cannot reach an agreement. The Preferred Reporting Items for Systematic review and Meta-Analyses (PRISMA) flow diagram will be used to present records of the selection process with clarifying the reason of exclusion.[42]

Data collection process

Data extraction

For peer review only

Two independent reviewers will review the full text of the included studies and extract the required data using a custom data extraction sheet. The third reviewer will be consulted if there is any disagreement between the two reviewers.

Dealing with missing data

The corresponding author of the included studies will be contacted if there is missing information.

Data items

The two reviewers will extract the following data from the included studies:

1. Bibliographic details: Study title, authors and year.
2. Study characteristics: Design, level of blinding and participants allocation.
3. Participants characteristics: Sample size, gender, age mean, handedness, stroke type, stroke location, time since stroke onset and severity of upper limb impairment.
4. The intervention group: tDCS montage, electrode size, intensity, density and duration, the technique of motor rehabilitation if conducted and the number of treatment sessions.
5. The control group: The type of intervention: sham tDCS plus motor rehabilitation or motor rehabilitation only.
6. Outcome measures: The fine dexterity measures and gross dexterity measures.
7. Results: The result of outcome of interest (improve, no change or deteriorate).

Outcome and prioritization

The primary outcomes of interest are fine dexterity measures and gross dexterity measures.

Risk of bias in individual studies

To discover any possibility of risk of bias within the included studies, the two reviewers will critically assess the methodological quality of the included studies using the Physiotherapy Evidence Database (PEDro) scale. The PEDro scale is a valid and reliable tool in critically appraising the randomised controlled trials.[43 44] It is comprised of 11 items and each one is scored either 1 if present or 0 if not. The total score is out of 10 because the first item is not included in the total score. After appraising the included studies, we will use the cutoff

For peer review only

points defined by Foley, Teasell, Bhogal et al.,[45] to classify each study according to PEDro score into one of the following: excellent quality (score 9 – 10), good quality (score 6 – 8), fair quality (score 4 – 5) and poor quality (score below 4).

Synthesis of results

The fine and gross dexterity measures will be analysed as continuous variables. We will obtain the sample size, mean and standard deviation (SD) for the baseline and post-intervention scores for intervention and control groups from each included study. We will contact the corresponding authors if there is missing data.

Data analysis

All data analysis will be conducted using the *R* software. First, we will quantify the effect size (the summary measure) for the difference between baseline and the post-intervention score of the outcome of interest for the intervention and control groups from each included study. Second, we will pool effect sizes in a meta-analysis in *R*. Meta-analysis will be performed using either a fixed- or random-effects model depending on the degree of heterogeneity. The results for fine and gross dexterity measures will be presented in two separate tables. The table includes the sample size, mean and SD for the baseline and post-intervention scores and the calculated effect size for the intervention and control groups.

Assessment of heterogeneity

We will use the I-squared (I^2) test to assess the heterogeneity statistically.[46] The I^2 is a common test being used in meta-analysis. It measures the variability percentage in the effect sizes caused by the variability between studies rather than sampling error.[46] Its score will be between 0% to 100%, with 0% reflecting no heterogeneity and 100% indicating very high heterogeneity.[47]

Meta-analysis

Meta-analysis will be performed using the fixed-effects model if the studies are similar ($I^2 < 25\%$).[48] If there is heterogeneity ($I^2 > 25\%$),[48] the random-effects model will be used. We will run a total of four main meta-analyses. First, we will assess the difference between intervention and control groups, with two separate meta-analyses conducted for studies

For peer review only - http://bmjopen.bmj.com/site/about/guidelines.xhtml

using fine compared to gross dexterity outcome measures. Second, we will evaluate the difference between baseline and the post-intervention across fine and gross dexterity outcome measures via two separate meta-analyses.

Additionally, we will run sub-group analyses to investigate the impact of patient characteristics on the effect of the tDCS. Specifically, we will split the participants according to severity of upper limb impairment (mild and moderate versus severe), stroke phase (sub-acute versus chronic) and stroke area (cortical versus subcortical) and run separate meta-analyses for each group. In case of considerable heterogeneity ($I^2 > 75\%$),[48] we will run subgroup analyses using the random-effects model to reveal which factors that underlie the variation between the studies. If the considerable variation between studies is persistent, the data will not be pooled in a meta-analysis. The result of the individual study will be interpreted narratively and presented in a summary table.

Meta-bias

Assessment of publication bias

Publication bias is the potential bias of publishing studies with positive results (rejecting the null hypothesis) rather than negative results,[49] causing a biased overestimate of the intervention effect. We will assess the possibility of publication bias in our study by creating funnel plots using *R*. Funnel plots are scatter plot showing the standard error (y-axis) against the effect size (x-axis) of the included studies. We will visually appraise the funnel plots to detect asymmetry, which implies the possibility of publication bias. Additionally, we will conduct Egger's test in *R* to quantitively assess asymmetry.[50]

Confidence in cumulative evidence

The overall quality of evidence of outcomes of interest of this study (fine and gross dexterity) will be judged by Grading of Recommendations, Assessment, Development and Evaluation (GRADE) approach.[51] The GRADE is a valid approach that produces implications of the evidence by evaluating the body of evidence at the outcome level rather than the study level. The assessment will be conducted separately for the fine dexterity and gross dexterity outcome measures across the following domains: risk of bias, publication bias and results consistency, directness and precision. Based on the overall GRADE score, the quality of

For peer review only

evidence will be rating one of the four levels: high (score ≥ 4), moderate (score = 3), low (score = 2) and very low (score ≤ 1).[51]

ETHICS AND DISSEMINATION

Ethical approval is not required for this study. The dissemination plan is to publish the results in a peer-review journal and presenting results in a conference.

PATIENT AND PUBLIC INVOLVEMENT

No patient involved.

AMENDMENTS

Protocol amendments will be documented in the final published paper.

AUTHORS' CONTRIBUTIONS

The protocol was substantially designed by LA and refined by NJ and LH. LH provided substantial guidance on analyses. LA drafted the manuscript. All authors edited the manuscript and read and approved the final version.

FUNDING STATEMENT

This study is a part of Lulwa Alabdulaali PhD, which is supported by a scholarship from Imam Abdulrahman Bin Faisal University, Saudi Arabia.

COMPETING INTERESTS STATEMENT

None declared.

For peer review only

REFERENCES

1. Adamson J, Beswick A, Ebrahim S. Is stroke the most common cause of disability? *Journal of stroke and cerebrovascular diseases* 2004;13(4):171-77.
2. Kwakkel G, Kollen BJ, van der Grond J, et al. Probability of regaining dexterity in the flaccid upper limb: impact of severity of paresis and time since onset in acute stroke. *Stroke* 2003;34(9):2181-86.
3. Lang CE, Wagner JM, Edwards DF, et al. Recovery of grasp versus reach in people with hemiparesis poststroke. *Neurorehabilitation and neural repair* 2006;20(4):444-54.
4. Canning CG, Ada L, O'Dwyer NJ. Abnormal muscle activation characteristics associated with loss of dexterity after stroke. *Journal of the neurological sciences* 2000;176(1):45-56.
5. Backman C, Gibson SCD, Parsons J. Assessment of hand function: the relationship between pegboard dexterity and applied dexterity. *Canadian Journal of Occupational Therapy* 1992;59(4):208-13.
6. Fleishman EA, Ellison GD. A factor analysis of fine manipulative tests. *Journal of Applied Psychology* 1962;46(2):96.
7. Kilbreath SL, Heard RC. Frequency of hand use in healthy older persons. *Australian Journal of Physiotherapy* 2005;51(2):119-22.
8. Nichols-Larsen DS, Clark P, Zeringue A, et al. Factors influencing stroke survivors' quality of life during subacute recovery. *Stroke* 2005;36(7):1480-84.
9. Lang CE, Wagner JM, Bastian AJ, et al. Deficits in grasp versus reach during acute hemiparesis. *Experimental Brain Research* 2005;166(1):126-36.
10. Platz T, Pinkowski C, van Wijck F, et al. Reliability and validity of arm function assessment with standardized guidelines for the Fugl-Meyer Test, Action Research Arm Test and Box and Block Test: a multicentre study. *Clinical rehabilitation* 2005;19(4):404-11.
11. Van der Lee JH, De Groot V, Beckerman H, et al. The intra-and interrater reliability of the action research arm test: a practical test of upper extremity function in patients with stroke. *Archives of physical medicine and rehabilitation* 2001;82(1):14-19.
12. Lefaucheur J-P, Antal A, Ayache SS, et al. Evidence-based guidelines on the therapeutic use of transcranial direct current stimulation (tDCS). *Clinical Neurophysiology* 2017;128(1):56-92.
13. Hsieh Y-w, Wu C-y, Lin K-c, et al. Responsiveness and validity of three outcome measures of motor function after stroke rehabilitation. *Stroke* 2009;40(4):1386-91.
14. Wolf SL, Catlin PA, Ellis M, et al. Assessing Wolf motor function test as outcome measure for research in patients after stroke. *Stroke* 2001;32(7):1635-39.
15. Rhee H-S, Kim S-J, Yu J-H. The effect of repetitive hand task on upper extremity proprioception and dexterity. *Journal of the Korean Society of Physical Medicine* 2010;5(4):685-92.
16. Rocha LSO, Gama GCB, Rocha RSB, et al. Constraint Induced Movement Therapy Increases Functionality and Quality of Life after Stroke. *Journal of Stroke and Cerebrovascular Diseases* 2021;30(6):105774.

For peer review only

17. Hummel F, Celnik P, Giraux P, et al. Effects of non-invasive cortical stimulation on skilled motor function in chronic stroke. *Brain* 2005;128(3):490-99.
18. Rocha S, Silva E, Foerster Á, et al. The impact of transcranial direct current stimulation (tDCS) combined with modified constraint-induced movement therapy (mCIMT) on upper limb function in chronic stroke: a double-blind randomized controlled trial. *Disability and rehabilitation* 2016;38(7):653-60.
19. Nitsche MA, Paulus W. Excitability changes induced in the human motor cortex by weak transcranial direct current stimulation. *The Journal of physiology* 2000;527(3):633-39.
20. Triccas LT, Burridge J, Hughes A, et al. Multiple sessions of transcranial direct current stimulation and upper extremity rehabilitation in stroke: a review and meta-analysis. *Clinical Neurophysiology* 2016;127(1):946-55.
21. Wu AD, Fregni F, Simon DK, et al. Noninvasive brain stimulation for Parkinson's disease and dystonia. *Neurotherapeutics* 2008;5(2):345-61.
22. Hummel FC, Heise K, Celnik P, et al. Facilitating skilled right hand motor function in older subjects by anodal polarization over the left primary motor cortex. *Neurobiology of aging* 2010;31(12):2160-68.
23. Bikson M, Grossman P, Thomas C, et al. Safety of transcranial direct current stimulation: evidence based update 2016. *Brain stimulation* 2016;9(5):641-61.
24. Traversa R, Cicinelli P, Pasqualetti P, et al. Follow-up of interhemispheric differences of motor evoked potentials from theaffected'andunaffected'hemispheres in human stroke. *Brain research* 1998;803(1-2):1-8.
25. Rehme AK, Eickhoff SB, Wang LE, et al. Dynamic causal modeling of cortical activity from the acute to the chronic stage after stroke. *Neuroimage* 2011;55(3):1147-58.
26. Murase N, Duque J, Mazzocchio R, et al. Influence of interhemispheric interactions on motor function in chronic stroke. *Annals of Neurology: Official Journal of the American Neurological Association and the Child Neurology Society* 2004;55(3):400-09.
27. Bai X, Guo Z, He L, et al. Different therapeutic effects of transcranial direct current stimulation on upper and lower limb recovery of stroke patients with motor dysfunction: a meta-analysis. *Neural plasticity* 2019;2019
28. Nitsche MA, Fricke K, Henschke U, et al. Pharmacological modulation of cortical excitability shifts induced by transcranial direct current stimulation in humans. *The Journal of physiology* 2003;553(1):293-301.
29. Hummel FC, Cohen LG. Drivers of brain plasticity. *Current opinion in neurology* 2005;18(6):667-74.
30. Bolognini N, Vallar G, Casati C, et al. Neurophysiological and behavioral effects of tDCS combined with constraint-induced movement therapy in poststroke patients. *Neurorehabilitation and neural repair* 2011;25(9):819-29.
31. Cunningham DA, Varnerin N, Machado A, et al. Stimulation targeting higher motor areas in stroke rehabilitation: A proof-of-concept, randomized, double-blinded placebo-controlled study of effectiveness and underlying mechanisms. *Restorative neurology and neuroscience* 2015;33(6):911-26.
32. Coscia M, Wessel MJ, Chaudary U, et al. Neurotechnology-aided interventions for upper limb motor rehabilitation in severe chronic stroke. *Brain* 2019;142(8):2182-97.
33. Beebe JA, Lang CE. Absence of a proximal to distal gradient of motor deficits in the upper extremity early after stroke. *Clinical Neurophysiology* 2008;119(9):2074-85.

For peer review only

34. Dum RP, Strick PL. Motor areas in the frontal lobe of the primate. *Physiology & behavior* 2002;77(4-5):677-82.
35. Lawrence DG, Kuypers HG. The functional organization of the motor system in the monkey: II. The effects of lesions of the descending brain-stem pathways. *Brain* 1968;91(1):15-36.
36. Schambra HM, Xu J, Branscheidt M, et al. Differential poststroke motor recovery in an arm versus hand muscle in the absence of motor evoked potentials. *Neurorehabilitation and neural repair* 2019;33(7):568-80.
37. Butler AJ, Shuster M, O'Hara E, et al. A meta-analysis of the efficacy of anodal transcranial direct current stimulation for upper limb motor recovery in stroke survivors. *Journal of Hand Therapy* 2013;26(2):162-71.
38. Bornheim S, Thibaut A, Beaudart C, et al. Evaluating the effects of tDCS in stroke patients using functional outcomes: a systematic review. *Disability and rehabilitation* 2020:1-11.
39. Kang N, Summers JJ, Cauraugh JH. Transcranial direct current stimulation facilitates motor learning post-stroke: a systematic review and meta-analysis. *Journal of Neurology, Neurosurgery & Psychiatry* 2016;87(4):345-55.
40. Marquez J, van Vliet P, McElduff P, et al. Transcranial direct current stimulation (tDCS): does it have merit in stroke rehabilitation? A systematic review. *International Journal of Stroke* 2015;10(3):306-16.
41. Shamseer L, Moher D, Clarke M, et al. Preferred reporting items for systematic review and meta-analysis protocols (PRISMA-P) 2015: elaboration and explanation. *Bmj* 2015;349
42. Page MJ, McKenzie JE, Bossuyt PM, et al. The PRISMA 2020 statement: an updated guideline for reporting systematic reviews. *Bmj* 2021;372
43. de Morton NA. The PEDro scale is a valid measure of the methodological quality of clinical trials: a demographic study. *Australian Journal of Physiotherapy* 2009;55(2):129-33.
44. Maher CG, Sherrington C, Herbert RD, et al. Reliability of the PEDro scale for rating quality of randomized controlled trials. *Physical therapy* 2003;83(8):713-21.
45. Foley NC, Teasell RW, Bhogal SK, et al. Stroke rehabilitation evidence-based review: methodology. *Topics in stroke rehabilitation* 2003;10(1):1-7.
46. Higgins JP, Thompson SG. Quantifying heterogeneity in a meta-analysis. *Statistics in medicine* 2002;21(11):1539-58.
47. Higgins JP, Thompson SG, Deeks JJ, et al. Measuring inconsistency in meta-analyses. *Bmj* 2003;327(7414):557-60.
48. Ried K. Interpreting and understanding meta-analysis graphs: a practical guide. 2006
49. Dickersin K. The existence of publication bias and risk factors for its occurrence. *Jama* 1990;263(10):1385-89.
50. Egger M, Smith GD, Schneider M, et al. Bias in meta-analysis detected by a simple, graphical test. *Bmj* 1997;315(7109):629-34.
51. Guyatt G, Oxman AD, Akl EA, et al. GRADE guidelines: 1. Introduction—GRADE evidence profiles and summary of findings tables. *Journal of clinical epidemiology* 2011;64(4):383-94.

For peer review only

SUPPLEMENTARY FILE 1:
Database search strategy for MEDLINE

1. Cerebrovascular accident
2. Cerebrovascular disorder
3. Cerebr*
4. Stroke
5. Poststroke
6. Post-stroke
7. CVA
8. Hemiplegi*
9. Hemipare*
10. Or/1,2,3,4,5,6,7,8,9
11. Transcranial direct current stimulation
12. TDCS
13. Brain stimulation
14. Or/11,12,13
15. Physiotherapy
16. Physical therapy
17. Occupational therapy
18. Rehabilitation
19. Motor training
20. Motor*
21. Exercise therapy
22. Constraint induced movement therapy
23. Modified constraint induced movement therapy
24. Or/15,16,17,18,19,20,21,22,23
25. Upper extremit*
26. Upper limb*
27. Arm
28. Hand
29. Or/25,26,27,28
30. Motor function
31. Function*
32. Functional outcome measure*
33. Outcome measure*
34. Dexterity
35. Dexter*
36. Manual dexterity
37. Fine
38. Gross
39. Or/30,31,32,33,34,35,36,37,38
40. Randomized controlled trial
41. RCT
42. Random*
43. Controlled clinical trial
44. Control*
45. Trial

For peer review only

46. Double-blind*
47. Double*
48. Sham-controlled
49. Sham*
50. Placebo-controlled
51. Placebo*
52. Groups
53. Allocate*
54. Assign*
55. Or/40,41,42,43,44,45,46,47,48,49,50,51,52,53,54
56. Child*
57. Cerebral palsy
58. Lower limb*
59. Lower extremit*
60. Leg
61. TMS
62. rTMS
63. Transcranial magnetic stimulation
64. Or/56,57,58,59,60,61,62,63
65. And/10,14,24,29,39,55
66. 65 NOT 64
67. Limit to human

For peer review only

PRISMA-P (Preferred Reporting Items for Systematic review and Meta-Analysis Protocols) 2015 checklist: recommended items to address in a systematic review protocol*

Section and topic	Item No	Checklist item
**ADMINISTRATIVE INFORMATION**		
Title:		
Identification	1a	Identify the report as a protocol of a systematic review
Update	1b	If the protocol is for an update of a previous systematic review, identify as such
Registration	2	If registered, provide the name of the registry (such as PROSPERO) and registration number
Authors:		
Contact	3a	Provide name, institutional affiliation, e-mail address of all protocol authors; provide physical mailing address of corresponding author
Contributions	3b	Describe contributions of protocol authors and identify the guarantor of the review
Amendments	4	If the protocol represents an amendment of a previously completed or published protocol, identify as such and list changes; otherwise, state plan for documenting important protocol amendments
Support:		
Sources	5a	Indicate sources of financial or other support for the review
Sponsor	5b	Provide name for the review funder and/or sponsor
Role of sponsor or funder	5c	Describe roles of funder(s), sponsor(s), and/or institution(s), if any, in developing the protocol
**INTRODUCTION**		
Rationale	6	Describe the rationale for the review in the context of what is already known
Objectives	7	Provide an explicit statement of the question(s) the review will address with reference to participants, interventions, comparators, and outcomes (PICO)
**METHODS**		
Eligibility criteria	8	Specify the study characteristics (such as PICO, study design, setting, time frame) and report characteristics (such as years considered, language, publication status) to be used as criteria for eligibility for the review
Information sources	9	Describe all intended information sources (such as electronic databases, contact with study authors, trial registers or other grey literature sources) with planned dates of coverage
Search strategy	10	Present draft of search strategy to be used for at least one electronic database, including planned limits, such that it could be repeated
Study records:		
Data management	11a	Describe the mechanism(s) that will be used to manage records and data throughout the review

For peer review only

Selection process	11b	State the process that will be used for selecting studies (such as two independent reviewers) through each phase of the review (that is, screening, eligibility and inclusion in meta-analysis)
Data collection process	11c	Describe planned method of extracting data from reports (such as piloting forms, done independently, in duplicate), any processes for obtaining and confirming data from investigators
Data items	12	List and define all variables for which data will be sought (such as PICO items, funding sources), any pre-planned data assumptions and simplifications
Outcomes and prioritization	13	List and define all outcomes for which data will be sought, including prioritization of main and additional outcomes, with rationale
Risk of bias in individual studies	14	Describe anticipated methods for assessing risk of bias of individual studies, including whether this will be done at the outcome or study level, or both; state how this information will be used in data synthesis
Data synthesis	15a	Describe criteria under which study data will be quantitatively synthesised
	15b	If data are appropriate for quantitative synthesis, describe planned summary measures, methods of handling data and methods of combining data from studies, including any planned exploration of consistency (such as I^2, Kendall's τ)
	15c	Describe any proposed additional analyses (such as sensitivity or subgroup analyses, meta-regression)
	15d	If quantitative synthesis is not appropriate, describe the type of summary planned
Meta-bias(es)	16	Specify any planned assessment of meta-bias(es) (such as publication bias across studies, selective reporting within studies)
Confidence in cumulative evidence	17	Describe how the strength of the body of evidence will be assessed (such as GRADE)

*** It is strongly recommended that this checklist be read in conjunction with the PRISMA-P Explanation and Elaboration (cite when available) for important clarification on the items. Amendments to a review protocol should be tracked and dated. The copyright for PRISMA-P (including checklist) is held by the PRISMA-P Group and is distributed under a Creative Commons Attribution Licence 4.0.**

From: Shamseer L, Moher D, Clarke M, Ghersi D, Liberati A, Petticrew M, Shekelle P, Stewart L, PRISMA-P Group. Preferred reporting items for systematic review and meta-analysis protocols (PRISMA-P) 2015: elaboration and explanation. BMJ. 2015 Jan 2;349(jan02 1):g7647.

For peer review only

BMJ Open

The effectiveness of transcranial direct current stimulation on hand dexterity in stroke patients: a protocol for a systematic review and meta-analysis.

Journal:	*BMJ Open*
Manuscript ID	bmjopen-2021-056064.R1
Article Type:	Protocol
Date Submitted by the Author:	10-Nov-2021

Complete List of Authors:	Alabdulaali, Lulwa; University of Birmingham; Imam Abdulrahman Bin Faisal University,<br>Hickman, Lydia; University of Birmingham, School of Psychology<br>Punt, T. David; University of Birmingham, School of Sport, Exercise and Rehabilitation Sciences<br>Jenkinson, Ned; University of Birmingham, School of Sport, Exercise and Rehabilitation Sciences; University of Birmingham, Centre for Human Brain Health

**Primary Subject Heading**:	Rehabilitation medicine
Secondary Subject Heading:	Rehabilitation medicine
Keywords:	REHABILITATION MEDICINE, Stroke < NEUROLOGY, NEUROLOGY

SCHOLARONE™
Manuscripts

TITLE: The effectiveness of transcranial direct current stimulation on hand dexterity in stroke patients: a protocol for a systematic review and meta-analysis.

AUTHORS

First author (corresponding author): Lulwa Alabdulaali

Institutional affiliation:

1- School of Sport, Exercise and Rehabilitation Sciences, University of Birmingham, UK
2- Department of Physical Therapy, College of Applied Medical Sciences, Imam Abdulrahman Bin Faisal University, Dammam, Saudi Arabia

e-mail address: Laa886@student.bham.ac.uk

Mailing address: University of Birmingham, Edgbaston, Birmingham, B15 2TT, UK

Second author: Lydia Hickman

Institutional affiliation:

1. School of Psychology, University of Birmingham, UK

e-mail address: Lxh856@student.bham.ac.uk

Third author: T. David Punt

Institutional affiliation:

1. School of Sport, Exercise and Rehabilitation Sciences, University of Birmingham, UK

e-mail address: T.D.Punt@bham.ac.uk

Fourth author: Ned Jenkinson

Institutional affiliation:

1. School of Sport, Exercise and Rehabilitation Sciences, University of Birmingham, UK
2. MRC-Versus Arthritis Centre for Musculoskeletal Ageing Research, University of Birmingham, UK
3. Centre for Human Brain Health, University of Birmingham, UK

e-mail address: n.jenkinson@bham.ac.uk

WORD COUNT: 3550

For peer review only - http://bmjopen.bmj.com/site/about/guidelines.xhtml

ABSTRACT

Introduction

Hand dexterity is the ability to execute the skilful movements using the hand and fingers. It is commonly impaired post-stroke resulting in a profound deterioration in the quality of life for stroke patients. Transcranial direct current stimulation (tDCS) is a form of non-invasive brain stimulation, which has gained a popularity as an adjunct therapy in recovering motor dysfunction post-stroke. Promising results have been gained from applying tDCS in combination with motor rehabilitation, however, the outcome of tDCS on the upper limb motor function post-stroke has been varied. Different results are potentially related to the discrepancy of the area of brain stimulation. Therefore, we aim to enhance the application of tDCS to improve its effectiveness in recovering hand dexterity through testing our hypothesis that stimulating the primary motor cortex could improve fine dexterity more than gross dexterity.

Methods and analysis

This protocol has been reported according to Preferred Reporting Items for Systematic Review and Meta-Analyses Protocols guidelines. CENTRAL, MEDLINE, EMBASE, SCOPUS, Web of Science and CINAHL databases will be searched with no restriction in language and publication date. The selected studies will be randomised controlled trial investigating the effect of tDCS alone or in combination with motor rehabilitation in improving hand dexterity of stroke patients with upper limb hemiparesis. The outcomes of interest are fine and gross hand dexterity measures. Two independent reviewers will assess the eligibility of the study, extract data and appraise the methodological quality. The data will be pooled in a meta-analysis if applicable or interpreted narratively. Grading of Recommendations, Assessment, Development and Evaluation (GRADE) approach will be used to assess the overall quality of evidence for the fine and gross dexterity measures.

Ethics and dissemination

Ethical approval is not required for this study. The dissemination plan is to publish the results in a peer-review journal and presenting results in a conference.

Registration details

The Protocol is registered in the International Prospective Register of Systematic Reviews database, under PROSPERO CRD42021262186.

For peer review only - http://bmjopen.bmj.com/site/about/guidelines.xhtml

STRENGTHS AND LIMITATIONS OF THIS STUDY

- This study will include the three stages of recovery of stroke patients.
- The search strategy designed to access both published and non-published studies.
- The search strategy is not limited to publication date or language.
- This study will be limited to the randomised controlled trials.

INTRODUCTION

Stroke is a common cause of disability in the United Kingdom.[1] Upper limb hemiparesis is a common impairment post-stroke, with seventy-six percent of stroke patients suffering from upper limb hemiparesis.[2] The clinical presentation of upper limb hemiparesis is muscle weakness and poor dexterity on the side of the body opposite of the side of brain lesion.[3 4] Dexterity refers to 'the fine, voluntary movements used to manipulate small objects during a specific task, as measured by the time required to complete the task'.[5(p209)] There are two types of dexterity: fine and gross. Fine dexterity is the ability to perform a delicate manipulation of very small object by precise movements of the fingertips.[6] Gross dexterity is the ability to make a manipulation of large object by coordinated arm-hand movement.[6] The majority of daily activities, such as self-care, dressing and eating, require a combination of fine and gross dexterity in combination with other upper limb movements.[7] Therefore, maintaining hand dexterity is an important determinant of quality of life for stroke patients.[8]

However, stroke patients with upper limb hemiparesis demonstrate dexterity dysfunction. They perform movements in a slow, uncoordinated manner because their hand movements lack accuracy and proper finger sequencing.[3 9] Likely more than half of the stroke patients have persistent hand dexterity deficiency at 6 months post stroke. Kwakkel and colleagues conducted a prospective cohort study,[2] in which they recruited 102 stroke patients from 7 different hospitals. All the patients were enrolled in an intensive rehabilitation program, with the study procedure starting within two weeks of stroke occurrence. Periodic follow up assessments of the upper and lower limbs function were conducted. For hand dexterity they used the Action Research Arm Test (ARAT), which is a reliable and valid tool for assessing hand function.[10 11] At first assessment, no dexterous hand movement was reported in the ARAT. At

For peer review only

6 months, 38% showed improvement in functional ability, while 62% persisted with loss of hand dexterity.[2]

Stroke rehabilitation involves assessing and improving hand dexterity in stroke patients in order to improve upper limb function.[12] Many functional assessment tools have been established to measure the level of upper limb motor function. These tools include tests for muscle strength and gross and fine hand dexterity. Examples of such tests include the Fugl Meyer Test,[13] Wolf Motor Function Test[14] and Action Research Arm Test.[10] There has also been considerable interest in examining the efficacy of different techniques to enhance upper limb function post-stroke, with some positive results described with a variety of methods as repetitive task training,[15] constraint induced movement therapy,[16] and non-invasive brain stimulation.[17 18]

A recent development has been the use of transcranial direct current stimulation (tDCS), a form of non-invasive brain stimulation. tDCS device includes a portable electrical current generator and two electrodes: cathode and anode, and works by delivering a low intensity constant electrical current to the brain that alters the cortical excitability.[19] It is a neuromodulatory technique that has gained popularity for use in recovering motor dysfunction and cognitive impairment.[12] It has been used to improve motor function among stroke patients, Parkinson's disease patients and old people.[17 20-22] Increased brain plasticity is thought to be the underlying mechanism of functional improvement.[19] Due to tDCS' promising effectiveness, cost-effectiveness, ease of application, safety, and tolerability, recent years have seen increased investigation of use.[12 19 23 24]

Studies exploring the effect of stroke on the brain using functional magnetic resonance imaging have observed decreased neuronal activity of the ipsilesional side few days post stroke.[25] This reduced activity, in turn decreases interhemispheric inhibition from the ipsilesional to the contralesional side, leading to an increase in contralesional neuronal activity, which increases interhemispheric inhibition from the contralesional to the ipsilesional side. This abnormal increase of interhemispheric inhibition likely restricts voluntary movement and therefore adversely affects motor recovery.[24-26] Given these abnormal patterns of activity, tDCS has been used in an attempt to produce focal changes in

For peer review only

neuronal activity to rebalance brain activity between the two hemispheres. The focal changes are either depolarising (diminishing the contralesional excitability), hyper-polarising (increasing the ipselisional excitability), or both, depending on the tDCS montage.[27 28]

Researchers have investigated factors to enhance the application of tDCS to increase its effectiveness, for example, the area of brain stimulation and the orientation of the electrodes.[19] There are three montages of tDCS application described in the literature in improving upper limb motor recovery post troke. The first montage is anodal tDCS. In this montage, the anodal electrode that produces a polarising effect is placed on the ipsilesional primary motor cortex (PMC), and the cathode electrode is used as a return electrode to close the electric circuit. It is placed on the contralesional supraorbital region.[12 19] In the cathodal tDCS montage, the cathode electrode that produces the depolarising effect is placed on the contralesional PMC. The anode electrode serves as the return electrode and is placed on the ipsilesional supraorbital region.[12 19] In the bilateral tDCS montage, the anodal electrode is placed over the ipsilesional PMC and the cathode is placed over the contralateral PMC.[12] Moreover, in controlled studies, tDCS can be applied as a sham stimulation. The sham tDCS is the method to blind the control group. The participant receives a very weak and short stimulating current, at a level below that which could produce any stimulation.[29]

tDCS has shown potential effectiveness in improving upper limb function post stroke when conducted both alone[22] or in combination with motor rehabilitation.[18 30] Several randomised controlled studies investigated the augmentation of the rehabilitation plasticity effect through conjunct therapy.[18 30 31] Conjunct therapy involves tDCS and motor rehabilitation. These studies found a superior beneficial effect of conjunct therapy in upper limb recovery compared to rehabilitation only.

RATIONALE

Given the essential role of the upper limbs in daily life, the recovery of upper limb motor function post stroke is an important issue for stroke rehabilitation community.[12] The efficacy of tDCS in improving hand function has been showed to be varied.[20] Discrepancies in results could be related to heterogeneity in the application of tDCS, e.g., the brain stimulating area.[12 32] The study done by Hummel, Celnik, Giraux et al.,[17] investigated the effect of stimulating the

For peer review only - http://bmjopen.bmj.com/site/about/guidelines.xhtml

primary motor cortex (PMC) using tDCS on upper limb function, using the Jebsen–Taylor Hand Function Test as an outcome measure. It includes subtests for both fine and gross hand dexterity. Hummel and colleagues found that the fine movements, like picking up paper clips, are more improved than gross movement like moving cans. This might be because the distal muscles of the upper limb receive higher input from the PMC (through the corticospinal tract) than the proximal muscles.[33]

Moreover, work from our laboratory[34 35] has shown a preferential effect of tDCS stimulating the PMC on movements of the hand as opposed to movements of the whole arm among healthy young and older adults. Given the PMC is the target of choice in studies investigating tDCS in upper limb recovery in stroke, this raises the question of whether a similar preferential recovery of fine versus gross hand dexterity is seen in these studies.

Therefore, we will conduct this systematic review and meta-analysis to enhance the tDCS application therefore optimising its therapeutic benefit in upper limb motor recovery post-stroke. Through investigating the difference in the recovery between fine and gross hand tasks. Specifically, we will investigate our hypothesis that stimulating the PMC using tDCS could improve the fine hand dexterity more than the gross dexterity because the fine hand tasks mainly require fingertip grasping while the gross hand tasks involve a combination of proximal arm movement with hand grasping.[6]

OBJECTIVES

This research aims to answer our research question “what is the difference in the tDCS effect between fine and gross hand dexterity of stroke patients with upper limb hemiparesis?”.

Specific objectives are:

1. To evaluate the effectiveness of tDCS on fine hand dexterity of stroke patients with upper limb hemiparesis.
2. To evaluate the effectiveness of tDCS on gross hand dexterity of stroke patients with upper limb hemiparesis.
3. To compare the effect of tDCS between fine and gross hand dexterity.
4. To investigate the impact of patient characteristics on the effect of tDCS.

For peer review only - http://bmjopen.bmj.com/site/about/guidelines.xhtml

METHODS AND ANALYSIS

This protocol has been reported according to Preferred Reporting Items for Systematic Review and Meta-Analyses Protocols (PRISMA-P) guidelines.[36]

Eligibility criteria

Types of studies

Inclusion criteria

- Randomised controlled double or single-blinded trials. Using either parallel or crossover design.
- English and non-English studies.
- Full-text studies.
- Published and unpublished studies.

Exclusion criteria

- Non-randomised clinical trial.
- Conference abstract.

Types of participants

The participants needed to be:

- Stroke patients aged 18 and above, both male and female with upper limb hemiparesis.
- Diagnosed with ischemic or haemorrhagic stroke, either cortical or subcortical.
- In the acute (1-7 days post-stroke) or sub-acute (7 days to 6 months post-stroke) or chronic phase (6 months or more post-stroke) when the intervention is applied.[37]
- Suffering from mild or moderate or severe upper limb impairment. The cut-off points of Fugl Meyer Assessment of the upper extremity (FMA-UE) will be used to classify the impairment into: severe (score 0-28), moderate (score 29-42), and mild (score 43 – 66).[38]

Types of intervention

Intervention group

For peer review only

The participants have received one or multiple sessions of tDCS that conducted alone or with motor rehabilitation. The tDCS either applied before or simultaneously with motor rehabilitation. The tDCS stimulated the primary motor cortex through one of the following montages: anodal, cathodal and bilateral. The motor rehabilitation includes any technique of motor training for upper limb.

Control group

The participants have received one or multiple sessions of sham tDCS or motor rehabilitation or sham tDCS plus motor rehabilitation.

For peer review only

Types of outcome measures

The outcomes of interest are as follow:

- Validated functional measures for fine hand dexterity; assess the ability to perform a delicate manipulation of very small object by precise movements of the fingertips.[6] For instance: Purdue Pegboard Test (PPT) and Nine Hole Peg Test (NHPT).
- Validated functional measures for gross hand dexterity; evaluates the ability to make a manipulation of large object by coordinated arm-hand movement.[6] For example: Box and Blocks Test (BBT) and Minnesota Manual Dexterity Test (MMDT).

Information sources

The study is planned to start on 1st August 2021 and the anticipated completion data of the study is 30th December 2021.

- We will conduct database and non-database searches with no restriction on publication date.

1. Database search

The following databases will be searched:

CENTRAL (Cochrane Central Register of Controlled Trials).

MEDLINE (Ovid).

EMBASE (Ovid).

SCOPUS.

Web of Science.

CINAHL (Cumulated Index of Nursing and Allied Health Literature).

2. Non-database search

For peer review only - http://bmjopen.bmj.com/site/about/guidelines.xhtml

We will review the reference list of the included studies.

- We will conduct grey literature search including ProQuest Dissertations and Theses, Open Access Theses and Dissertations (OATD), and Electronic Thesis Online Service (EThOS) databases.

Search strategy

An example of search strategy for searching MEDLINE is available as an online supplementary file 1.

Study records

Data management

The search records from the selected databases including the study title, abstract will be exported to a reference management software (EndNote X9). The EndNote X9 will organise the records and recognise the duplicate studies.

Selection process

After removing the duplicate reports using EndNote X9, two independent reviewers will assess the eligibility of the study and discard the irrelevant ones. Initially, the two reviewers will review the studies title and abstract according to the inclusion criteria and exclude the irrelevant studies. Then the full text will be obtained and screened for those studies that potentially meet the inclusion criteria and if more details are required to confirm study exclusion. The two reviewers will decide about the study inclusion and will make discussion if there is disagreement. The third reviewer will make the final decision if the two reviewers cannot reach an agreement. The Preferred Reporting Items for Systematic review and Meta-Analyses (PRISMA) flow diagram will be used to present records of the selection process with clarifying the reason of exclusion.[39]

Data collection process

Data extraction

Two independent reviewers will review the full text of the included studies and extract the required data using a custom data extraction sheet. The third reviewer will be consulted if there is any disagreement between the two reviewers.

For peer review only - http://bmjopen.bmj.com/site/about/guidelines.xhtml

Dealing with missing data

The corresponding author of the included studies will be contacted if there is missing information.

Data items

The two reviewers will extract the following data from the included studies:

1. Bibliographic details: Study title, authors and year.
2. Study characteristics: Design, level of blinding and participants allocation.
3. Participants characteristics: Sample size, gender, age mean, handedness, stroke type, stroke location, time since stroke onset and severity of upper limb impairment.
4. The intervention group: tDCS montage, electrode size, intensity, density and duration, the technique of motor rehabilitation if conducted and the number of treatment sessions.
5. The control group: The type of intervention: sham tDCS plus motor rehabilitation or motor rehabilitation only.
6. Outcome measures: The fine dexterity measures and gross dexterity measures.
7. Results: The result of outcome of interest (improve, no change or deteriorate).

Outcome and prioritization

The primary outcomes of interest are fine dexterity measures and gross dexterity measures.

Risk of bias in individual studies

To discover any possibility of risk of bias within the included studies, the two reviewers will critically assess the methodological quality of the included studies using the Physiotherapy Evidence Database (PEDro) scale. The PEDro scale is a valid and reliable tool in critically appraising the randomised controlled trials.[40 41] It is comprised of 11 items and each one is scored either 1 if present or 0 if not. The total score is out of 10 because the first item is not included in the total score. After appraising the included studies, we will use the cutoff points defined by Foley, Teasell, Bhogal et al.,[42] to classify each study according to PEDro score into one of the following: excellent quality (score 9 – 10), good quality (score 6 – 8), fair quality (score 4 – 5) and poor quality (score below 4).

For peer review only - http://bmjopen.bmj.com/site/about/guidelines.xhtml

Synthesis of results

The fine and gross dexterity measures will be analysed as continuous variables. The analysis will be based on within-subject comparisons for both parallel and crossover design RCTs. We will obtain the sample size, mean and standard deviation (SD) for the baseline and post-intervention scores of the fine and gross dexterity measures for both the intervention and control groups from each included study. We will contact the corresponding authors if there is missing data.

Data analysis

All data analysis will be conducted using the *R* software. First, we will quantify the Cohen's d effect size for the difference between baseline and the post-intervention score of the fine and gross dexterity measures for the intervention and control groups from each included study. Second, we will calculate pooled effect sizes and confidence intervals of various groups of studies via meta-analyses in *R*. We will display separate tables for each meta-analysis containing the corresponding sample sizes and effect sizes of the studies included in the sample.

Assessment of heterogeneity

We will use the I-squared (I^2) test to assess the heterogeneity statistically.[43] The I^2 is a common test being used in meta-analysis. It measures the variability percentage in the effect sizes caused by the variability between studies rather than sampling error.[43] Its score will be between 0% to 100%, with 0% reflecting no heterogeneity and 100% indicating very high heterogeneity.[44]

Meta-analysis

Meta-analysis will be performed using the fixed-effects model if the studies are similar ($I^2 <$ 25%).[45] If there is heterogeneity ($I^2 >$ 25%),[45] the random-effects model will be used. We will run a total of four main meta-analyses:

1. Fine dexterity meta-analysis (intervention group)
2. Fine dexterity meta-analysis (control group)
3. Gross dexterity meta-analysis (intervention group)

For peer review only

4. Gross dexterity meta-analysis (control group)

To make comparisons between the outcomes of each meta-analysis, we will inspect the pooled effects sizes and identify whether there are overlapping confidence intervals. If the confidence intervals overlap, we will conclude that there is no difference between the pooled effect sizes. However, if the confidence intervals do not overlap, we will conclude that there is a significant difference between the pooled effect sizes.

First, we will assess the treatment effect of the tDCS on fine dexterity through comparing the pooled effect sizes of fine dexterity meta-analysis (meta-analysis 1) and control of fine dexterity meta-analysis (meta-analysis 2). Second, we will evaluate the treatment effect of the tDCS on gross dexterity through comparing the pooled effect sizes of gross dexterity meta-analysis (meta-analysis 3) and control of gross dexterity meta-analysis (meta-analysis 4). Third, we will compare the effect of tDCS between fine and gross dexterity through comparing the pooled effect sizes of fine dexterity meta-analysis (meta-analysis 1) and gross dexterity meta-analysis (meta-analysis 3).

Additionally, we will run exploratory sub-group analyses to investigate the impact of patient characteristics on the effect of the tDCS. Specifically, we will split the participants according to severity of upper limb impairment (mild and moderate versus severe), stroke phase (acute and sub-acute versus chronic) and stroke area (cortical versus subcortical) and run separate meta-analyses for each group. Then we will compare the pooled effect sizes.

In case of considerable heterogeneity ($I^2 > 75\%$),[45] we will run subgroup analyses using the random-effects model to reveal which factors that underlie the variation between the studies. If the considerable variation between studies is persistent, the data will not be pooled in a meta-analysis. The result of the individual study will be interpreted narratively and presented in a summary table.

Meta-bias

Assessment of publication bias

For peer review only - http://bmjopen.bmj.com/site/about/guidelines.xhtml

Publication bias is the potential bias of publishing studies with positive results (rejecting the null hypothesis) rather than negative results,[46] causing a biased overestimate of the intervention effect. We will assess the possibility of publication bias in our study by creating funnel plots using *R*. Funnel plots are scatter plot showing the standard error (y-axis) against the effect size (x-axis) of the included studies. We will visually appraise the funnel plots to detect asymmetry, which implies the possibility of publication bias. Additionally, we will conduct Egger's test in *R* to quantitively assess asymmetry.[47]

Confidence in cumulative evidence

The overall quality of evidence of outcomes of interest of this study (fine and gross dexterity) will be judged by Grading of Recommendations, Assessment, Development and Evaluation (GRADE) approach.[48] The GRADE is a valid approach that produces implications of the evidence by evaluating the body of evidence at the outcome level rather than the study level. The assessment will be conducted separately for the fine dexterity and gross dexterity outcome measures across the following domains: risk of bias, publication bias and results consistency, directness and precision. Based on the overall GRADE score, the quality of evidence will be rating one of the four levels: high (score $\geq$ 4), moderate (score = 3), low (score = 2) and very low (score $\leq$ 1).[48]

Discussion

Several systematic reviews have been conducted to assess the effect of tDCS on upper limb motor function.[20 27 49-52] These previous reviews reviewed the impact of tDCS on improving upper limb functional outcomes. However, they considered the improvement in the upper limb, with no differentiation between fine and gross hand dexterity. As such, this review and meta-analysis will look for the difference between tDCS effect on fine and gross hand dexterity. Therefore, while numerous tools are available for assessing upper limb function, the outcomes of this study will be limited to reliable and valid fine and gross dexterity measures. Because several of the available tools cannot be considered a test of fine or gross dexterity only, they include subtests that assess different aspects of upper limb motor function like joints movements, muscle strength, and hand dexterity without a split into fine and gross dexterity subtests.

For peer review only

The current study will include crossover design RCTs. A common concern with crossover trials is the carryover effect that occurs when the treatment effect of the first period of the study carries over to the next period, which could affect the participant's response.[53] While the washout period between the two treatment period could reduce the carryover effect, the crossover design might be inappropriate to investigate the intervention with long lasting effect.[53] However, the effect of a single session of the tDCS persists for several minutes up to one hour after the stimulation.[19 54] Therefore, the crossover design is widely used in tDCS studies because the possibility of the carryover effect is very low.

ETHICS AND DISSEMINATION

Ethical approval is not required for this study. The dissemination plan is to publish the results in a peer-review journal and presenting results in a conference.

PATIENT AND PUBLIC INVOLVEMENT

No patient involved.

AMENDMENTS

Protocol amendments will be documented in the final published paper.

AUTHORS' CONTRIBUTIONS

The protocol was substantially designed by LA and refined by NJ, LH and TP. LH provided substantial guidance on analyses. LA drafted the manuscript. All authors edited the manuscript and read and approved the final version.

FUNDING STATEMENT

This study is a part of Lulwa Alabdulaali PhD, which is supported by a scholarship from Imam Abdulrahman Bin Faisal University, Saudi Arabia.

COMPETING INTERESTS STATEMENT

None declared.

For peer review only

REFERENCES

1. Adamson J, Beswick A, Ebrahim S. Is stroke the most common cause of disability? *Journal of stroke and cerebrovascular diseases* 2004;13(4):171-77.
2. Kwakkel G, Kollen BJ, van der Grond J, et al. Probability of regaining dexterity in the flaccid upper limb: impact of severity of paresis and time since onset in acute stroke. *Stroke* 2003;34(9):2181-86.
3. Lang CE, Wagner JM, Edwards DF, et al. Recovery of grasp versus reach in people with hemiparesis poststroke. *Neurorehabilitation and neural repair* 2006;20(4):444-54.
4. Canning CG, Ada L, O'Dwyer NJ. Abnormal muscle activation characteristics associated with loss of dexterity after stroke. *Journal of the neurological sciences* 2000;176(1):45-56.
5. Backman C, Gibson SCD, Parsons J. Assessment of hand function: the relationship between pegboard dexterity and applied dexterity. *Canadian Journal of Occupational Therapy* 1992;59(4):208-13.
6. Fleishman EA, Ellison GD. A factor analysis of fine manipulative tests. *Journal of Applied Psychology* 1962;46(2):96.
7. Kilbreath SL, Heard RC. Frequency of hand use in healthy older persons. *Australian Journal of Physiotherapy* 2005;51(2):119-22.
8. Nichols-Larsen DS, Clark P, Zeringue A, et al. Factors influencing stroke survivors' quality of life during subacute recovery. *Stroke* 2005;36(7):1480-84.
9. Lang CE, Wagner JM, Bastian AJ, et al. Deficits in grasp versus reach during acute hemiparesis. *Experimental Brain Research* 2005;166(1):126-36.
10. Platz T, Pinkowski C, van Wijck F, et al. Reliability and validity of arm function assessment with standardized guidelines for the Fugl-Meyer Test, Action Research Arm Test and Box and Block Test: a multicentre study. *Clinical rehabilitation* 2005;19(4):404-11.
11. Van der Lee JH, De Groot V, Beckerman H, et al. The intra-and interrater reliability of the action research arm test: a practical test of upper extremity function in patients with stroke. *Archives of physical medicine and rehabilitation* 2001;82(1):14-19.
12. Lefaucheur J-P, Antal A, Ayache SS, et al. Evidence-based guidelines on the therapeutic use of transcranial direct current stimulation (tDCS). *Clinical Neurophysiology* 2017;128(1):56-92.
13. Hsieh Y-w, Wu C-y, Lin K-c, et al. Responsiveness and validity of three outcome measures of motor function after stroke rehabilitation. *Stroke* 2009;40(4):1386-91.
14. Wolf SL, Catlin PA, Ellis M, et al. Assessing Wolf motor function test as outcome measure for research in patients after stroke. *Stroke* 2001;32(7):1635-39.
15. Rhee H-S, Kim S-J, Yu J-H. The effect of repetitive hand task on upper extremity proprioception and dexterity. *Journal of the Korean Society of Physical Medicine* 2010;5(4):685-92.
16. Rocha LSO, Gama GCB, Rocha RSB, et al. Constraint Induced Movement Therapy Increases Functionality and Quality of Life after Stroke. *Journal of Stroke and Cerebrovascular Diseases* 2021;30(6):105774.
17. Hummel F, Celnik P, Giraux P, et al. Effects of non-invasive cortical stimulation on skilled motor function in chronic stroke. *Brain* 2005;128(3):490-99.
18. Rocha S, Silva E, Foerster Á, et al. The impact of transcranial direct current stimulation (tDCS) combined with modified constraint-induced movement therapy (mCIMT) on

For peer review only - http://bmjopen.bmj.com/site/about/guidelines.xhtml

upper limb function in chronic stroke: a double-blind randomized controlled trial. *Disability and rehabilitation* 2016;38(7):653-60.
19. Nitsche MA, Paulus W. Excitability changes induced in the human motor cortex by weak transcranial direct current stimulation. *The Journal of physiology* 2000;527(3):633-39.
20. Triccas LT, Burridge J, Hughes A, et al. Multiple sessions of transcranial direct current stimulation and upper extremity rehabilitation in stroke: a review and meta-analysis. *Clinical Neurophysiology* 2016;127(1):946-55.
21. Wu AD, Fregni F, Simon DK, et al. Noninvasive brain stimulation for Parkinson's disease and dystonia. *Neurotherapeutics* 2008;5(2):345-61.
22. Hummel FC, Heise K, Celnik P, et al. Facilitating skilled right hand motor function in older subjects by anodal polarization over the left primary motor cortex. *Neurobiology of aging* 2010;31(12):2160-68.
23. Bikson M, Grossman P, Thomas C, et al. Safety of transcranial direct current stimulation: evidence based update 2016. *Brain stimulation* 2016;9(5):641-61.
24. Traversa R, Cicinelli P, Pasqualetti P, et al. Follow-up of interhemispheric differences of motor evoked potentials from theaffected'andunaffected'hemispheres in human stroke. *Brain research* 1998;803(1-2):1-8.
25. Rehme AK, Eickhoff SB, Wang LE, et al. Dynamic causal modeling of cortical activity from the acute to the chronic stage after stroke. *Neuroimage* 2011;55(3):1147-58.
26. Murase N, Duque J, Mazzocchio R, et al. Influence of interhemispheric interactions on motor function in chronic stroke. *Annals of Neurology: Official Journal of the American Neurological Association and the Child Neurology Society* 2004;55(3):400-09.
27. Bai X, Guo Z, He L, et al. Different therapeutic effects of transcranial direct current stimulation on upper and lower limb recovery of stroke patients with motor dysfunction: a meta-analysis. *Neural plasticity* 2019;2019
28. Nitsche MA, Fricke K, Henschke U, et al. Pharmacological modulation of cortical excitability shifts induced by transcranial direct current stimulation in humans. *The Journal of physiology* 2003;553(1):293-301.
29. Hummel FC, Cohen LG. Drivers of brain plasticity. *Current opinion in neurology* 2005;18(6):667-74.
30. Bolognini N, Vallar G, Casati C, et al. Neurophysiological and behavioral effects of tDCS combined with constraint-induced movement therapy in poststroke patients. *Neurorehabilitation and neural repair* 2011;25(9):819-29.
31. Cunningham DA, Varnerin N, Machado A, et al. Stimulation targeting higher motor areas in stroke rehabilitation: A proof-of-concept, randomized, double-blinded placebo-controlled study of effectiveness and underlying mechanisms. *Restorative neurology and neuroscience* 2015;33(6):911-26.
32. Coscia M, Wessel MJ, Chaudary U, et al. Neurotechnology-aided interventions for upper limb motor rehabilitation in severe chronic stroke. *Brain* 2019;142(8):2182-97.
33. Turton A, Lemon R. The contribution of fast corticospinal input to the voluntary activation of proximal muscles in normal subjects and in stroke patients. *Experimental Brain Research* 1999;129(4):559-72.
34. Weightman M, Brittain J-S, Punt D, et al. Targeted tDCS selectively improves motor adaptation with the proximal and distal upper limb. *Brain stimulation* 2020;13(3):707-16.

For peer review only

35. Weightman M, Brittain J-S, Miall RC, et al. Direct and indirect effects of cathodal cerebellar TDCS on visuomotor adaptation of hand and arm movements. *Scientific Reports* 2021;11(1):1-10.
36. Shamseer L, Moher D, Clarke M, et al. Preferred reporting items for systematic review and meta-analysis protocols (PRISMA-P) 2015: elaboration and explanation. *Bmj* 2015;349
37. Stinear CM, Lang CE, Zeiler S, et al. Advances and challenges in stroke rehabilitation. *The Lancet Neurology* 2020;19(4):348-60.
38. Woytowicz EJ, Rietschel JC, Goodman RN, et al. Determining levels of upper extremity movement impairment by applying a cluster analysis to the Fugl-Meyer assessment of the upper extremity in chronic stroke. *Archives of physical medicine and rehabilitation* 2017;98(3):456-62.
39. Page MJ, McKenzie JE, Bossuyt PM, et al. The PRISMA 2020 statement: an updated guideline for reporting systematic reviews. *Bmj* 2021;372
40. de Morton NA. The PEDro scale is a valid measure of the methodological quality of clinical trials: a demographic study. *Australian Journal of Physiotherapy* 2009;55(2):129-33.
41. Maher CG, Sherrington C, Herbert RD, et al. Reliability of the PEDro scale for rating quality of randomized controlled trials. *Physical therapy* 2003;83(8):713-21.
42. Foley NC, Teasell RW, Bhogal SK, et al. Stroke rehabilitation evidence-based review: methodology. *Topics in stroke rehabilitation* 2003;10(1):1-7.
43. Higgins JP, Thompson SG. Quantifying heterogeneity in a meta-analysis. *Statistics in medicine* 2002;21(11):1539-58.
44. Higgins JP, Thompson SG, Deeks JJ, et al. Measuring inconsistency in meta-analyses. *Bmj* 2003;327(7414):557-60.
45. Ried K. Interpreting and understanding meta-analysis graphs: a practical guide. 2006
46. Dickersin K. The existence of publication bias and risk factors for its occurrence. *Jama* 1990;263(10):1385-89.
47. Egger M, Smith GD, Schneider M, et al. Bias in meta-analysis detected by a simple, graphical test. *Bmj* 1997;315(7109):629-34.
48. Guyatt G, Oxman AD, Akl EA, et al. GRADE guidelines: 1. Introduction—GRADE evidence profiles and summary of findings tables. *Journal of clinical epidemiology* 2011;64(4):383-94.
49. Butler AJ, Shuster M, O'Hara E, et al. A meta-analysis of the efficacy of anodal transcranial direct current stimulation for upper limb motor recovery in stroke survivors. *Journal of Hand Therapy* 2013;26(2):162-71.
50. Bornheim S, Thibaut A, Beaudart C, et al. Evaluating the effects of tDCS in stroke patients using functional outcomes: a systematic review. *Disability and rehabilitation* 2020:1-11.
51. Kang N, Summers JJ, Cauraugh JH. Transcranial direct current stimulation facilitates motor learning post-stroke: a systematic review and meta-analysis. *Journal of Neurology, Neurosurgery & Psychiatry* 2016;87(4):345-55.
52. Marquez J, van Vliet P, McElduff P, et al. Transcranial direct current stimulation (tDCS): does it have merit in stroke rehabilitation? A systematic review. *International Journal of Stroke* 2015;10(3):306-16.
53. Elbourne DR, Altman DG, Higgins JP, et al. Meta-analyses involving cross-over trials: methodological issues. *International journal of epidemiology* 2002;31(1):140-49.

For peer review only - http://bmjopen.bmj.com/site/about/guidelines.xhtml

54. Nitsche MA, Nitsche MS, Klein CC, et al. Level of action of cathodal DC polarisation induced inhibition of the human motor cortex. *Clinical Neurophysiology* 2003;114(4):600-04.

For peer review only

SUPPLEMENTARY FILE 1:
Database search strategy for MEDLINE

1. Cerebrovascular accident
2. Cerebrovascular disorder
3. Cerebr*
4. Stroke
5. Poststroke
6. Post-stroke
7. CVA
8. Hemiplegi*
9. Hemipare*
10. Or/1,2,3,4,5,6,7,8,9
11. Transcranial direct current stimulation
12. TDCS
13. Brain stimulation
14. Or/11,12,13
15. Physiotherapy
16. Physical therapy
17. Occupational therapy
18. Rehabilitation
19. Motor training
20. Motor*
21. Exercise therapy
22. Constraint induced movement therapy
23. Modified constraint induced movement therapy
24. Or/15,16,17,18,19,20,21,22,23
25. Upper extremit*
26. Upper limb*
27. Arm
28. Hand
29. Or/25,26,27,28
30. Motor function
31. Function*
32. Functional outcome measure*
33. Outcome measure*
34. Dexterity
35. Dexter*
36. Manual dexterity
37. Fine
38. Gross
39. Or/30,31,32,33,34,35,36,37,38
40. Randomized controlled trial
41. RCT
42. Random*
43. Controlled clinical trial
44. Control*
45. Trial

For peer review only

46. Double-blind*
47. Double*
48. Sham-controlled
49. Sham*
50. Placebo-controlled
51. Placebo*
52. Groups
53. Allocate*
54. Assign*
55. Or/40,41,42,43,44,45,46,47,48,49,50,51,52,53,54
56. Child*
57. Cerebral palsy
58. Lower limb*
59. Lower extremit*
60. Leg
61. TMS
62. rTMS
63. Transcranial magnetic stimulation
64. Or/56,57,58,59,60,61,62,63
65. And/10,14,24,29,39,55
66. 65 NOT 64
67. Limit to human

For peer review only

PRISMA-P (Preferred Reporting Items for Systematic review and Meta-Analysis Protocols) 2015 checklist: recommended items to address in a systematic review protocol*

Section and topic	Item No	Checklist item	Page number
**ADMINISTRATIVE INFORMATION**			
Title:			1
Identification	1a	Identify the report as a protocol of a systematic review	1
Update	1b	If the protocol is for an update of a previous systematic review, identify as such	NA
Registration	2	If registered, provide the name of the registry (such as PROSPERO) and registration number	2
Authors:			1
Contact	3a	Provide name, institutional affiliation, e-mail address of all protocol authors; provide physical mailing address of corresponding author	
Contributions	3b	Describe contributions of protocol authors and identify the guarantor of the review	1, 14
Amendments	4	If the protocol represents an amendment of a previously completed or published protocol, identify as such and list changes; otherwise, state plan for documenting important protocol amendments	NA
Support:			
Sources	5a	Indicate sources of financial or other support for the review	14
Sponsor	5b	Provide name for the review funder and/or sponsor	14
Role of sponsor or funder	5c	Describe roles of funder(s), sponsor(s), and/or institution(s), if any, in developing the protocol	NA
**INTRODUCTION**			
Rationale	6	Describe the rationale for the review in the context of what is already known	5, 6
Objectives	7	Provide an explicit statement of the question(s) the review will address with reference to participants, interventions, comparators, and outcomes (PICO)	6
**METHODS**			
Eligibility criteria	8	Specify the study characteristics (such as PICO, study design, setting, time frame) and report characteristics (such as years considered, language, publication status) to be used as criteria for eligibility for the review	7, 8
Information sources	9	Describe all intended information sources (such as electronic databases, contact with study authors, trial registers or other grey literature sources) with planned dates of coverage	8, 9
Search strategy	10	Present draft of search strategy to be used for at least one electronic database, including planned limits, such that it could be repeated	Supplementary file 1
Study records:			

For peer review only

Data management	11a	Describe the mechanism(s) that will be used to manage records and data throughout the review	9
Selection process	11b	State the process that will be used for selecting studies (such as two independent reviewers) through each phase of the review (that is, screening, eligibility and inclusion in meta-analysis)	9
Data collection process	11c	Describe planned method of extracting data from reports (such as piloting forms, done independently, in duplicate), any processes for obtaining and confirming data from investigators	9, 10
Data items	12	List and define all variables for which data will be sought (such as PICO items, funding sources), any pre-planned data assumptions and simplifications	10
Outcomes and prioritization	13	List and define all outcomes for which data will be sought, including prioritization of main and additional outcomes, with rationale	10
Risk of bias in individual studies	14	Describe anticipated methods for assessing risk of bias of individual studies, including whether this will be done at the outcome or study level, or both; state how this information will be used in data synthesis	10
Data synthesis	15a	Describe criteria under which study data will be quantitatively synthesised	11
	15b	If data are appropriate for quantitative synthesis, describe planned summary measures, methods of handling data and methods of combining data from studies, including any planned exploration of consistency (such as I^2, Kendall's τ)	11, 12
	15c	Describe any proposed additional analyses (such as sensitivity or subgroup analyses, meta-regression)	12
	15d	If quantitative synthesis is not appropriate, describe the type of summary planned	12
Meta-bias(es)	16	Specify any planned assessment of meta-bias(es) (such as publication bias across studies, selective reporting within studies)	12, 13
Confidence in cumulative evidence	17	Describe how the strength of the body of evidence will be assessed (such as GRADE)	13

*** It is strongly recommended that this checklist be read in conjunction with the PRISMA-P Explanation and Elaboration (cite when available) for important clarification on the items. Amendments to a review protocol should be tracked and dated. The copyright for PRISMA-P (including checklist) is held by the PRISMA-P Group and is distributed under a Creative Commons Attribution Licence 4.0.**

From: Shamseer L, Moher D, Clarke M, Ghersi D, Liberati A, Petticrew M, Shekelle P, Stewart L, PRISMA-P Group. Preferred reporting items for systematic review and meta-analysis protocols (PRISMA-P) 2015: elaboration and explanation. BMJ. 2015 Jan 2;349(jan02 1):g7647.

For peer review only
